# Advanced Functional Tumor Imaging and Precision Nuclear Medicine Enabled by Digital PET Technologies

**DOI:** 10.1155/2017/5260305

**Published:** 2017-05-16

**Authors:** Chadwick L. Wright, Katherine Binzel, Jun Zhang, Michael V. Knopp

**Affiliations:** Wright Center of Innovation in Biomedical Imaging, Department of Radiology, The Ohio State University Wexner Medical Center, 395 W. 12th Avenue, Rm. 430, Columbus, OH 43210, USA

## Abstract

The purpose of this article is to provide a brief overview of the background, basic principles, technological evolution, clinical capabilities, and future directions for functional tumor imaging as PET evolves from the conventional photomultiplier tube-based platform into a fully digital detector acquisition platform. The recent introduction of solid-state digital photon counting PET detector is the latest evolution of clinical PET which enables faster time-of-flight timing resolution that leads to more precise localization of the annihilation events and further contributes to reduction in partial volume and thus makes high definition and ultrahigh definition PET imaging feasible with current standard acquisition procedures. The technological advances of digital PET can be further leveraged by optimizing many of the acquisition and reconstruction methodologies to achieve faster image acquisition to improve cancer patient throughput, lower patient dose in accordance with ALARA, and improved quantitative accuracy to enable biomarker capability. Digital PET technology will advance molecular imaging capabilities beyond oncology and enable Precision Nuclear Medicine.

## 1. Introduction

The purpose of this article is to briefly introduce the current technological evolution that is enabled by digital detector technology and discuss its applicability to clinical positron emission tomography/computed tomography (PET/CT). Although PET/CT utilization has been primarily oncologic, functional molecular imaging has the opportunity for expanded utilization in both oncologic and nononcologic applications and will demand reduced ionizing radiotracer doses and improved quantification. The paradigm of Precision Nuclear Medicine incorporates new strategies to advance functional molecular imaging with more detailed visualization and more robust quantification of disease burden. These aspects are essential both for diagnostic and for therapy management opportunities including the further evolution as validated biomarkers. Even today, there are considerable unmet clinical needs such as the frequently observed indeterminate PET lesion, detectability of subcentimeter lesions, and lack of biomarker validation for response assessment. While many technologies have been rapidly moving away from cathode-ray tubes and analog signal processing, nuclear medicine and PET are still predominantly using the analog photomultiplier tube acquisition technologies. The recent introduction of solid-state detectors is a transformative technology change for clinical nuclear medicine. As with many other technology changes, solid-state and digital acquisition technologies can be implemented in different ways. One major driving force to pursue the replacement of the photomultiplier tube technology was the goal of integrated PET/magnetic resonance imaging (MRI) and next-generation PET/CT systems. While the acquisition chain consists of multiple components, especially the crystal characteristics, this article highlights the clinical opportunities enabled by this change of the detector technology. The most recent technology advance for PET/CT has been the clinical demonstration of a solid-state system which also has the best clinical system time-of-flight timing capabilities. This article summarizes our initial experiences with a focus on demonstrating the overall opportunities supported by next-generation digital PET technology.

## 2. Basic Principles of PET Detector Technology

**Figure 1:**
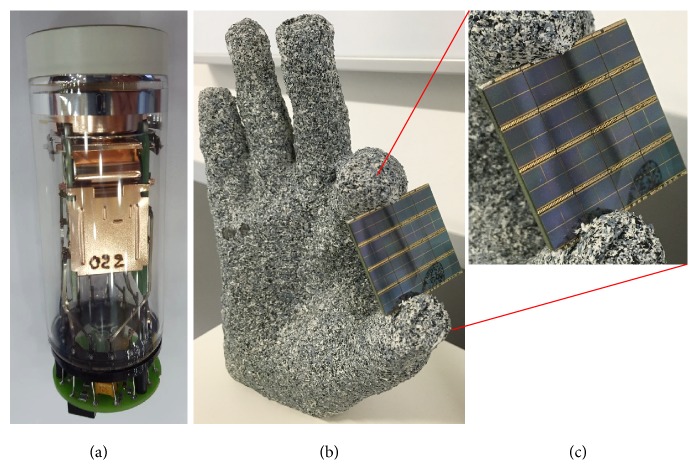
The photomultiplier tube detector unit from a cPET system (a) which has recently been replaced with a solid-state DPC PET detector unit (b) in the next-generation dPET/CT system. (c) A closer view of the DPC PET detector (Philips Healthcare, Cleveland, Ohio, USA). The DPC PET detector unit enables fully digital 1 : 1 coupling with the scintillation crystals within the dPET detector ring assembly.

Photomultiplier tube (PMT) detectors have been used since the early days of PET [1] and have not changed fundamentally except for manufacturing and timing improvements. As a PMT cannot operate within a strong magnetic field, solid-state avalanche photodiodes (APD) and silicon photomultipliers (SiPM) were developed to operate within such fields [2–4]. Initially, solid-state APD/SiPM detectors used analog signal processing approaches to translate photon detection into quantifiable annihilation events. The next leap in solid-state detectors was the introduction of digital photon counting (DPC) which eliminates any analog-to-digital conversion and thus enables preferential signal characteristics and speed [5].  Figure 1 shows a PMT unit from a conventional PET (cPET) system and the new DPC detector from digital PET (dPET) system. Combining these capabilities with direct one-to-one coupling to the unchanged detection crystals eliminates the need for Anger logic that was essential to estimate the localization of a photon event when the detector is significantly larger than the crystal to which it is coupled. Over the last 10 years, the timing resolution has become of increasing relevance as the benefits of time-of-flight (ToF) information for the more precise localization of the annihilation event in its linear trajectory led to improved lesion detectability [6]. While there are many other factors in the digital signal processing, this article will highlight the advancements of going from analog to solid-state digital processing and the potential applications for functional tumor imaging.

## 3. Clinical Evolution of PET Detector Technology

Whole-body PET became clinically feasible in the late 1980s and subsequently evolved from a 2D to a 3D multiring platform [1]. The next leap was the creation of a multimodal approach with the introduction of hybrid PET/CT systems around the change of the millennium. Later on in that decade, ToF became clinically available and further leveraged the 3D multiring platform [7]. Over the years, the *z*-axis coverage increased as a means of faster whole-body acquisition or larger organ coverage. While dynamic acquisitions were part of the early evolution of PET, it became unsupported during the focus of hybrid whole-body imaging but was rejuvenated in the last decade.

With the growth of MRI and the superb soft tissue contrast achievable, the vision of hybrid PET/MR systems rapidly evolved in the last decade [3, 4]. The facilitating technology for PET/MR was the availability of MRI-compatible APD/SiPM PET detectors which still relied upon Anger logic and analog signal processing just like the PMT-based systems that preceded it. One of the early limitations of this initial solid-state technology was the absence of ToF capability for clinical imaging. At present, current generation PET/MR systems support ToF timing resolution around 400 ps [8].

The initial lack of full digitization in the SiPM detectors led to the next technological leap with the introduction of digital photon counting (DPC) detector technology [5, 9–14]. Combining this with a direct one-to-one coupling streamlines the signal processing. This currently represents the most advanced dPET detector technology which has been introduced into the next-generation clinical dPET/CT [15–17] and preclinical dPET/MR [18, 19] systems.

## 4. Features of PET Systems

Here, we briefly present 7 features of clinical PET systems that are particularly relevant for understanding the clinical implications of new PET detector technologies [17].

### 4.1. Spatial Resolution

Spatial resolution of a PET scanner is an intrinsic feature of the detector chain which reflects the ability of the system to physically differentiate between two sources within the minimum distance between two points in a reconstructed image [1]. The physical size of the crystal element usually plays a dominant role in determining spatial resolution for PET. The fundamental limits for spatial resolution are also determined by contributions from positron range, noncollinearity, placement of detectors, decoding errors, systems noise, and reconstruction methodologies which may limit or degrade the effective resolution of the PET system [1, 20]. While these also apply to dPET systems, innovative designs such as one-to-one coupling significantly improved ToF timing resolution, and PSF-integrated reconstruction methodologies facilitate improvements [16]. In summary, dPET systems do not necessarily have improved physical spatial resolution, which is predominantly influenced by the crystal size; however, these systems contribute to improved clinical imaging characteristics due to the above advancements.

### 4.2. Sensitivity

Sensitivity of a PET system represents the ability to detect the true annihilation event rate. It is normally expressed in counts per unit time per unit of activity present in a source and depends on factors like solid angle, system photon detection efficiency, and dead time. Sensitivity in PET was substantially improved with the introduction of 3D acquisition; however, the sensitivity profile usually degrades from a peak in the center to both edges. In particular, 3D PET acquisitions have a rapid decrease in linear sensitivity due to poor counting statistics at the edges, thus requiring an overlap in longitudinal field of view between adjacent bed positions. Similarly, sensitivity decreases linearly within the axial field of view. An early observation and key advantage of the dPET detector technology is its virtually zero dead time, improving dPET system sensitivity [16]. This is particularly important for clinical studies which routinely administer radiotracer doses that generate count rates which exceed those used for typical PET scanner characterization (e.g., NEMA NU-2).

### 4.3. Noise Equivalent Count Rate (NECR)

Image noise of a PET system is usually characterized by the NECR which is substantially improved with dPET systems [21]. In our initial experience with a precommercial release dPET/CT system, we observed 156% improvement at ~51 kBq/mL when compared with an existing PMT-based cPET system [17].

### 4.4. Image Acquisition

One goal to advance clinical nuclear medicine is to image faster and thus reduce patient motion, patient discomfort, table time, and the need for sedation/anesthesia [15]. One clinically relevant approach is to invest the gains of dPET detector sensitivity and precision into reducing the image acquisition time for static, whole-body, and dynamic dPET imaging. This potential for faster dPET image acquisition helps to minimize patient motion-based artifacts.

### 4.5. Image Reconstruction

Despite many technological advances in PET, today's clinical PET imaging reconstruction approaches utilize matrix sizes smaller than or equal to 200 and voxel lengths of 3-4 mm [22]. CT and MRI have increased their reconstruction matrix sizes due to improvements in signal generation which all led to advances in image quality. Therefore, dPET is poised to similarly embrace its ability to improve image reconstruction and visualization of more precisely detected annihilation events. This is a major area of opportunity to leverage dPET technology and therefore we have proposed refined nomenclature to characterize reconstructed PET images into* standard definition* (SD, matrix size ≤ 200),* high definition* (HD, matrix size > 200 but ≤ 400), and* ultrahigh definition* (UHD, matrix size > 400) [23]. Furthermore, HD and UHD image reconstruction utilizes PSF and Gaussian filtering as part of its overall optimization. These advances contribute to better visualization of the more precisely detected PET events [15, 17].

### 4.6. Time-of-Flight

The clinical benefit of ToF has been well recognized in the recent years [24]; however, the timing resolution of cPET systems was still limited to about 500 ps or greater [7, 25]. The dPET technologies facilitate substantially improved timing resolutions of 400 ps and better [8, 16, 17]. Ongoing clinical trials are evaluating whether this improvement in timing resolution may lead to improved lesion detectability and more precise quantification. The initial experience using a dPET/CT system with ToF capability of 325 ps indicates in phantoms that those expectations can be met and may lead to meaningful clinical improvements [15].

### 4.7. Radiotracer Dose

Another approach for better utilizing the higher sensitivity and precision of the dPET detector technology is to substantially reduce radiotracer dosing [15, 17, 21]. Radiation dose reduction is a key enabler to expand the clinical utilization of advanced functional molecular imaging methodologies like PET for clinical response assessment in patients undergoing therapeutic interventions as well as nononcologic clinical applications. This is another major area of opportunity to benefit from dPET technology and we have proposed refined nomenclature to characterize the PET dosing level for patients. Here is a proposed approach for ^18^F-FDG oncologic whole-body PET imaging:* standard dose* (S_DOSE_, ^18^F-FDG ≥ 370 MBq but < 740 MBq),* low dose* (L_DOSE_, ^18^F-FDG ≥ 185 MBq but < 370 MBq),* ultralow dose* (UL_DOSE_, ^18^F-FDG ≥ 37 MBq but < 185 MBq), and* super-ultralow dose* (SUL_DOSE_, ^18^F-FDG < 37 MBq).

In summary, it has to be highlighted that the dPET technology enables the refinement of many different components that impact overall image quality, lesion detectability, and quantification.

## 5. Emerging Concepts for Functional Tumor Imaging Enabled by dPET

Our team has extensive experience performing more than 150 intraindividual comparison studies between dPET/CT and cPET/CT systems currently focusing on using standard of care, standard dose, and standard definition imaging [15]. The new dPET system technology has been performing well with consistent timing resolution better than 325 ps and excellent system stability for over 16 months [17].

### 5.1. Improved Lesion Detectability Enabled by Higher Definition Visualization

**Figure 2:**
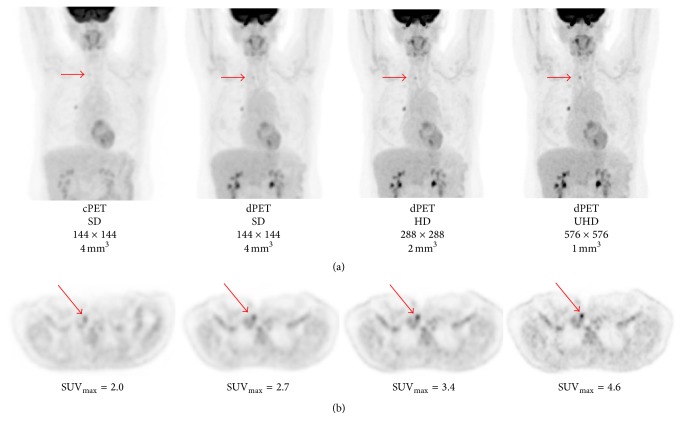
Intraindividual comparison in a patient scanned on the cPET/CT (Gemini 64 ToF, Philips Healthcare, Cleveland, Ohio, USA) system and a precommercial release dPET/CT (Vereos, Philips Healthcare, Cleveland, Ohio, USA) system using different reconstruction matrix/voxel volume sizes with a 3D line-of-response TOF blob-based algorithm [5, 17, 22, 23]. The patient was intravenously given a standard dose of 485 MBq of ^18^F-FDG and then underwent imaging on the dPET/CT system at 50 min and the cPET/CT system at 76 min after injection. Both cPET and dPET emission scans were acquired with 90 s per bed position. Although there is a discrete ^18^F-FDG-avid lesion noted in the right hilar region on both cPET and dPET images, there is a subcentimeter lesion in the right supraclavicular region which is only apparent on dPET images and becomes more conspicuous (and more suspicious) with higher definition image reconstructions. (a) Maximum intensity projection images from standard definition cPET (matrix size = 144 × 144, voxel volume = 4 mm^3^), standard definition dPET (144 × 144, 4 mm^3^), high definition dPET (288 × 288, 2 mm^3^), and ultrahigh definition dPET (576 × 576, 1 mm^3^). Point spread function and Gaussian filtering were applied to both high definition and ultrahigh definition dPET reconstructed images but not to standard definition dPET or cPET images. (b) Axial images from standard definition cPET, standard definition dPET, high definition dPET, and ultrahigh definition dPET taken at the level of the lesion in the right supraclavicular region. Region-of-interest analysis of the right supraclavicular lesion demonstrates FDG avidity similar to background on the cPET whereas the conspicuity and SUV_max_ values increase with higher definition dPET. This case illustrates the capability of dPET technology to substantially improve lesion detectability, lesion characterization, and diagnostic confidence.

Digital PET has the ability to use larger reconstruction matrices with smaller voxel volumes which enables a more robust visualization of smaller metabolically active lesions. Currently, most cPET images are reconstructed using standard definition matrix sizes of 144–200. We anticipate that dPET imaging will routinely use high definition reconstruction with matrix sizes between 200 and 400 while using unchanged acquisition times. Initial results indicate that even ultrahigh definition imaging with matrix sizes greater than 400 can be readily accomplished for current whole-body imaging protocols leading to voxel volumes comparable to CT and/or MRI [15, 17].  Figure 2 illustrates the potential for improved lesion detectability enabled with dPET/CT using standard and higher definition reconstructions. Decreasing the voxel volume increases the visual conspicuity of lesions due to the substantially reduced partial volume and thus leads to higher definition image quality [22]. This improves lesion detectability without any apparent increase in background tissue uptake.

### 5.2. Faster Image Acquisition and/or Lower Radiotracer Dose Imaging

Based on the improved sensitivity and precision of the dPET detector platform, there is potential for enabling faster whole-body PET image acquisitions [15]. With list-mode acquisition, the possibility exists for simulating shorter frame durations through data clipping. Our initial dPET observations indicate that a reduction of image acquisition times by more than 50% appears to be feasible without impacting image quality and/or quantification at current standard dosing levels for FDG. Alternatively, the capabilities of dPET can also be used to reduce radiotracer dose [21] while maintaining standard acquisition times. A combination which shortens acquisition time and reduces dose is also readily feasible, dependent upon individual imaging needs. In reality, dPET enables the opportunity to advance image quality, reduce acquisition time, and lower the dose compared to current standard-of-care PET approaches.

### 5.3. Improved Recovery Coefficient and Its Impact on Quantification

A well-established limitation of cPET is the deterioration of the recovery coefficient as lesion size decreases which weakens the quantitative precision for response assessment. This challenge not only affects small lesions but also affects quantitative precision when evaluating heterogeneous lesions. Although recovery coefficient and quantification are impacted by many components including detector characteristics, count density, timing resolution of ToF, and reconstruction approach, dPET technology has the potential to advance the quantitative precision for smaller and heterogeneous lesions in order to facilitate more consistency across multisite and multisystem clinical trials (e.g., EARL harmonization). It has been demonstrated that dPET has the highest overall system performance with consistently improved recovery coefficients when compared with cPET [17].

## 6. Future Directions

**Figure 3:**
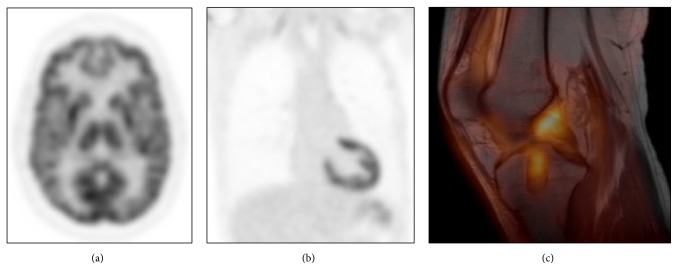
Nononcologic clinical opportunities for lower dose/higher definition imaging enabled by next-generation dPET include (a) neurologic, (b) cardiovascular, and (c) orthopedic/sports medicine indications. Digital PET cases demonstrated were imaged using standard ^18^F-FDG doses of 448 MBq and 477 MBq for (a) and (b), respectively, and ultralow ^18^F-FDG dose of 100 MBq for (c). The dPET acquisitions were obtained at 55 min after injection for (a), 53 min after injection for (b), and 60 min after injection for (c). The dPET emission scans were acquired with 90 s per bed position for (a) and (b) but (c) was a limited single bed acquisition for 15 min. Low dose CT attenuation scans were acquired using 120 kV and 50 mA with dose modulation and using iterative iDose^4^ reconstruction.

The current vision for the use of digital PET technology is either to implement it within hybrid MR systems or to improve the existing diagnostic/therapy management capabilities of PET/CT systems. It is our opinion that the DPC technology is truly the next generation in the evolution of PET imaging systems both as hybrid PET/CT and as PET/MR. The technological advances can be further leveraged by optimizing many of the PET acquisition and reconstruction methodologies to achieve disease-specific and organization-specific goals (e.g., faster image acquisition to improve patient throughput, lower patient dose in accordance with “as low as reasonably achievable” (ALARA), and improved quantitative accuracy to enable biomarker capability). PET image quality has not fundamentally changed over the last two decades and is poised to leap forward with high definition and even ultrahigh definition imaging. If we enable a substantial reduction in radiotracer dose, we have an opportunity to utilize PET more broadly in nononcologic applications (Figure 3) such as neuroscience, cardiovascular disease, sports medicine, and inflammation imaging. All of these benefits are very synergistic with the development of new PET radiotracers or new applications for existing radiotracers. The further evolution of clinical PET/MR will certainly benefit from the broader adoption of DPC detector technology as evidenced by the recent development of preclinical prototypes and our initial clinical evidence that dPET enables improved lesion detectability, lesion characterization, and diagnostic confidence.

## 7. Conclusion

This article highlights the fundamental technology innovations that led to the current development of next-generation digital PET systems. The wider clinical availability of dPET may be the inflection point to move clinical PET practice beyond oncology and into other nononcologic molecular imaging applications. In summary, digital PET is a transformative technology that will advance the paradigm of Precision Nuclear Medicine to address the unmet clinical needs for better tumor lesion detectability, improved lesion characterization especially for indeterminate lesions, more rapid biomarker validation for therapy response assessment, and radiotracer dose reduction in accordance with ALARA.

## Abbreviations

^18^F-FDG: Fluorine-18-fluorodeoxyglucose
ALARA: As low as reasonably achievable
APD: Avalanche photodiode
CT: Computed tomography
cPET: Conventional positron emission tomography
DPC: Digital photon counting
dPET: Digital positron emission tomography
FWHM: Full width half max
MR: Magnetic resonance
MRI: Magnetic resonance imaging
PET: Positron emission tomography
PMT: Photomultiplier tube
PSF: Point spread function
SiPM: Silicon photomultiplier.
